# The *Znt7*-null mutation has sex dependent effects on the gut microbiota and goblet cell population in the mouse colon

**DOI:** 10.1371/journal.pone.0239681

**Published:** 2020-09-29

**Authors:** Mary E. Kable, Niknaz Riazati, Catherine P. Kirschke, Junli Zhao, Surapun Tepaamorndech, Liping Huang

**Affiliations:** 1 Immunity and Disease Prevention Research Unit, USDA-ARS, Western Human Nutrition Research Center, Davis, California, United States of America; 2 Department of Nutrition, University of California Davis, Davis, California, United States of America; 3 Obesity and Metabolism Research Unit, USDA-ARS, Western Human Nutrition Research Center, Davis, California, United States of America; 4 School of Food Science, Nanjing Xiaozhuang University, Nanjing, Jiangsu, China; 5 Food Biotechnology Research Unit, National Center for Genetic Engineering and Biotechnology (BIOTEC), Khlong Luang, Pathum Thani, Thailand; National Institute for Research on Agriculture, Alimentation and Environment (INRAE), FRANCE

## Abstract

Cellular homeostasis of zinc, an essential element for living organisms, is tightly regulated by a family of zinc transporters. The zinc transporter 7, ZnT7, is highly expressed on the membrane of the Golgi complex of intestinal epithelial cells and goblet cells. It has previously been shown that *Znt7* knockout leads to zinc deficiency and decreased weight gain in C57BL/6 mice on a defined diet. However, effects within the colon are unknown. Given the expression profile of *Znt7*, we set out to analyze the changes in mucin density and gut microbial composition in the mouse large intestine induced by *Znt7* knockout. We fed a semi-purified diet containing 30 mg Zn/kg to *Znt7*^*-/-*^ mice with their heterozygous and wild type littermates and found a sex specific effect on colonic mucin density, goblet cell number, and microbiome composition. In male mice *Znt7* knockout led to increased goblet cell number and mucin density but had little effect on gut microbiome composition. However, in female mice *Znt7* knockout was associated with decreased goblet cell number and mucin density, with increased proportions of the microbial taxa, *Allobaculum*, relative to wild type. The gut microbial composition was correlated with mucin density in both sexes. These findings suggest that a sex-specific relationship exists between zinc homeostasis, mucin production and the microbial community composition within the colon.

## Introduction

Zinc is an essential trace metal for living organisms. In mammals, cellular zinc homeostasis is primarily maintained by two families of zinc transporters; ZnT (zinc transporter, SLC30A family, 10 members), and ZIP (Zrt- and Irt-related protein, SLC39A family, 14 members). It has been demonstrated that the majority of zinc transporter proteins function in control of cellular zinc homeostasis [1, 2]. However, some zinc transporter proteins, such as ZIP8 and ZnT10, also transport manganese across the cell membrane [3, 4]. The primary function of the ZIP proteins is to bring zinc into the cytoplasm either from the extracellular space or intracellular storage in order to maintain cellular zinc homeostasis. On the other hand, ZnT proteins control zinc homeostasis, with the exception of ZnT1, a zinc exporter [5], by importing zinc into intracellular organelles for storage, secretion, or integration into metalloproteins [6]. Members of the two zinc transporter families display unique expression patterns in tissues and cell types, though overlapping expression within zinc transporter families has also been detected (https://www.ebi.ac.uk/gxa/home).

ZnT7, a zinc transporter responsible for zinc accumulation in the Golgi apparatus of the cell [7, 8], is highly expressed in absorptive epithelial cells as well as goblet cells in the mouse gastrointestinal tract [9, 10]. In the large intestine, the number of goblet cells is much higher than in the small intestine [11]. These cells secrete mucins that form a gel-like barrier on the epithelial surface of the gut. Mucins play an important role in health. They contribute to the absorption of zinc [12], protection of the gut epithelium from commensal microbes and pathogenic microorganisms [13–16], and motility of ingested foods and feces along the upper and lower gastrointestinal tract. It has been shown that knockout of the *Znt7* gene (*Znt7*-KO) negatively affects zinc absorption in the mouse gut causing zinc deficiency [9], which is evident by lower serum zinc concentrations and lower cellular zinc content in many tissues, including small intestine, liver, kidney, and bone. However, the mechanism for decreased absorption of dietary zinc in the gut of *Znt7* knockout mice relative to wild type (WT) mice has not yet been fully elucidated. It is possible that reduced accumulation of zinc in the Golgi apparatus of the enterocytes and goblet cells lining the gut, affects the health of these cells, which in turn affects the mucin composition, gut microbiota composition, and absorptive capacity of the intestinal lining.

This hypothesis is supported by previous evidence describing the role of zinc in shaping the environment of the intestine. Animal and *in vitro* studies have shown that zinc deficiency can decrease overall bacterial species richness, reduce predicted levels of bacterial genes related to nutrient acquisition [17] and impair intestinal integrity through decreased expression of the tight junction proteins, occludin and claudin [17–19]. Additionally, a higher dietary zinc requirement for conventionally-raised rats relative to germ-free controls indicates that zinc is essential for the growth of microbial organisms within the gut [20]. Together, these studies suggest that zinc homeostasis plays roles in both the intestinal barrier function and microbial composition within the gut.

We hypothesized that the absence of ZnT7 would alter cellular zinc homeostasis in goblet cells in the gut, leading to changes in gut mucosal and microbial composition. Hence, we examined the effects of *Znt7* knockout in mice fed a semi-purified diet with a defined amount of dietary zinc (30 mg Zn/kg diet, a zinc adequate diet for rodents) on mucin production and the gut microbiota in the colon. We compared goblet cell numbers, quantities of mucins associated with goblet cells, and the gut microbiota from male and female mice in three study groups (WT, *Znt7*^+/-^ and *Znt7*^-/-^). After a 4-week feeding (6–10 weeks of age), *Znt7*^*-/-*^ mice weighed significantly less than the WT controls in both male and female groups. Interestingly, changes in goblet cell numbers, mucin density, and gut microbiota composition were sex specific.

## Results

### *Znt7* knockout in mice reduced body weight

**Fig 1:**
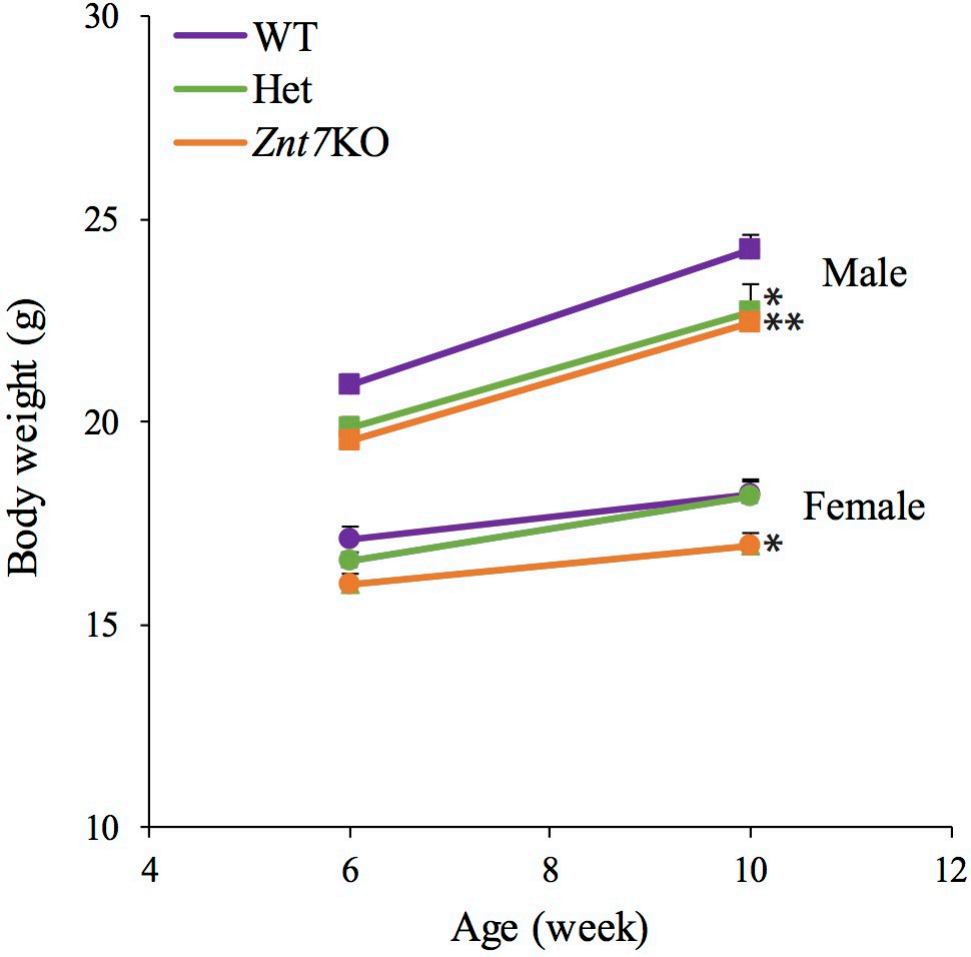
*Znt7*^*-/-*^ mice show reduced body weight after 4 weeks on a semi-purified diet (30 mg/kg Zn). Offspring, including *Znt7*^-/-^, *Znt7*^+/-^, and WT produced from *Znt7*^+/-^ breeding pairs, were fed a semi-purified rodent diet containing 30 mg Zn/kg ad libitum between 6- and 10-weeks of age. Body weights obtained at 6- and 10-weeks of age are shown. All values are expressed as mean±S.E. (*error bars*), n = 6-12/group. *, *p*<0.05 (to the WT control in the same sex group); **, *p*<0.01 (to the WT control in the same sex group). WT, wild type control mice; Het, heterozygous *Znt7* knockout mice; *Znt7*KO, homozygous *Znt7* knockout mice.

In the current study, body weights of male and female mice of the three genotypes (WT, *Znt7*^+/-^ and *Znt7*^-/-^) were recorded at 6 and 10 weeks of age, representing the point at which a defined diet with 30 mg Zn/kg was introduced and 4 weeks after introduction of the diet. After 4 weeks on a defined diet, the male WT control weighed an average of 24.2±0.4 g (n = 12) while male *Znt7*^+/-^ and *Znt7*^-/-^ mice weighed 22.7±0.7 g (n = 9) and 22.5±0.3 g (n = 6), respectively (Fig 1). The difference in body weights between male WT and *Znt7*^+/-^ or *Znt7*^-/-^ mice was statistically significant (*p*<0.05, WT vs *Znt7*^+/-^ (6.4±0.9%); *p*<0.01, WT vs *Znt7*^-/-^ (7.4±1.2%)). The average weights for females at 10-week old were 18.2±0.3 g for WT (n = 6), 18.2±0.2 g for *Znt7*^+/-^ (n = 13), and 17.0±0.3 g for *Znt7*^-/-^ mice (n = 9). Female *Znt7*^-/-^ mice weighed significantly less (7.0±0.5%, *p*<0.05) than WT (Fig 1). Together, these growth results are consistent with our previously published data [9]. *Znt7* allelic deficiency had a negative effect on the growth of male mice but not females.

### Reduced goblet cell numbers and goblet cell-associated mucin levels in the colon of female *Znt7*^-/-^ mice

**Fig 2:**
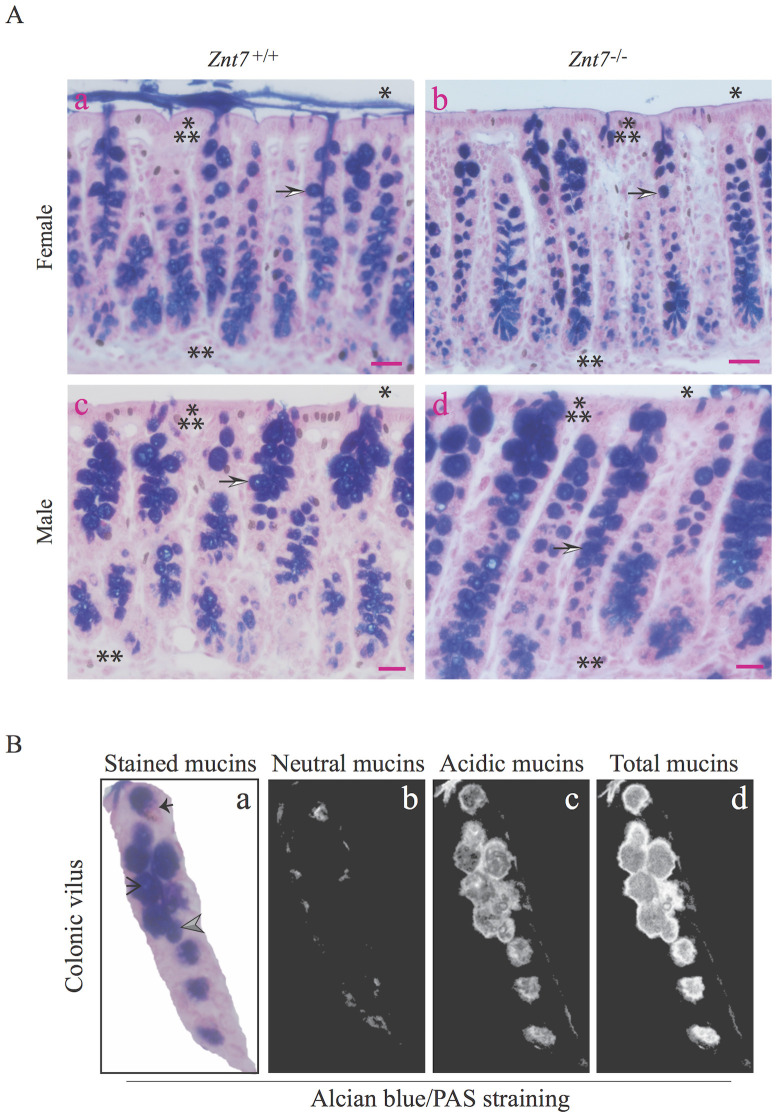
Mucin staining and quantification in the mouse colon. (A) Representative staining from the colon tissue (1 cm distal to the cecum) isolated from (a) female WT, (b) female *Znt7*^-/-^, (c) male WT, and (d) male *Znt7*^-/-^ mice. The sections were stained with Alcian blue/PAS and counterstained with Nuclear Fast Red to visualize cell nuclei. Arrows indicate goblet cells. * indicates the lumen side of the colon and ** indicate the basal side of the colon. ⁂ indicates the epithelium of the villus. Scale bar = 25 μm. (B) Representative images for automated quantification of color shades in the colonic villus using TissueQuant software. (a) An Alcian blue/PAS stained image. The solid and open arrows indicate neutral and acidic mucins stained magenta and bright blue, respectively. The gray arrow denotes the overlapping of neutral and acidic mucins (dark blue/purple). TissueQuant masking for (b) magenta color shade for neutral mucins, (c) blue color shade for acidic mucins, and (d) dark blue/purple color shade for both neutral and acidic mucins.

In order to understand the effect of *Znt7* knockout on mucin contents in the colon, we performed Alcian blue/PAS staining where Alcian blue stained acidic mucins blue and PAS stained neutral mucins magenta (Fig 2A). Total mucin intensities in goblet cells were determined in the colonic tissue sections prepared from WT and *Znt7*^-/-^ mice (both male and female; n = 5-6/genotype/sex; n = 18 villi/mouse) and analyzed using TissueQuant software [21]. ‘TissueQuant’ stored the color shades of interest (blue, magenta) in gray files (Fig 2B). Then, the algorithm assigned smaller scores to paler shades and larger scores to deeper shades with a zero number for any other color shade. So that, the blue and magenta could be quantified with pixels of the color shades.

**Fig 3:**
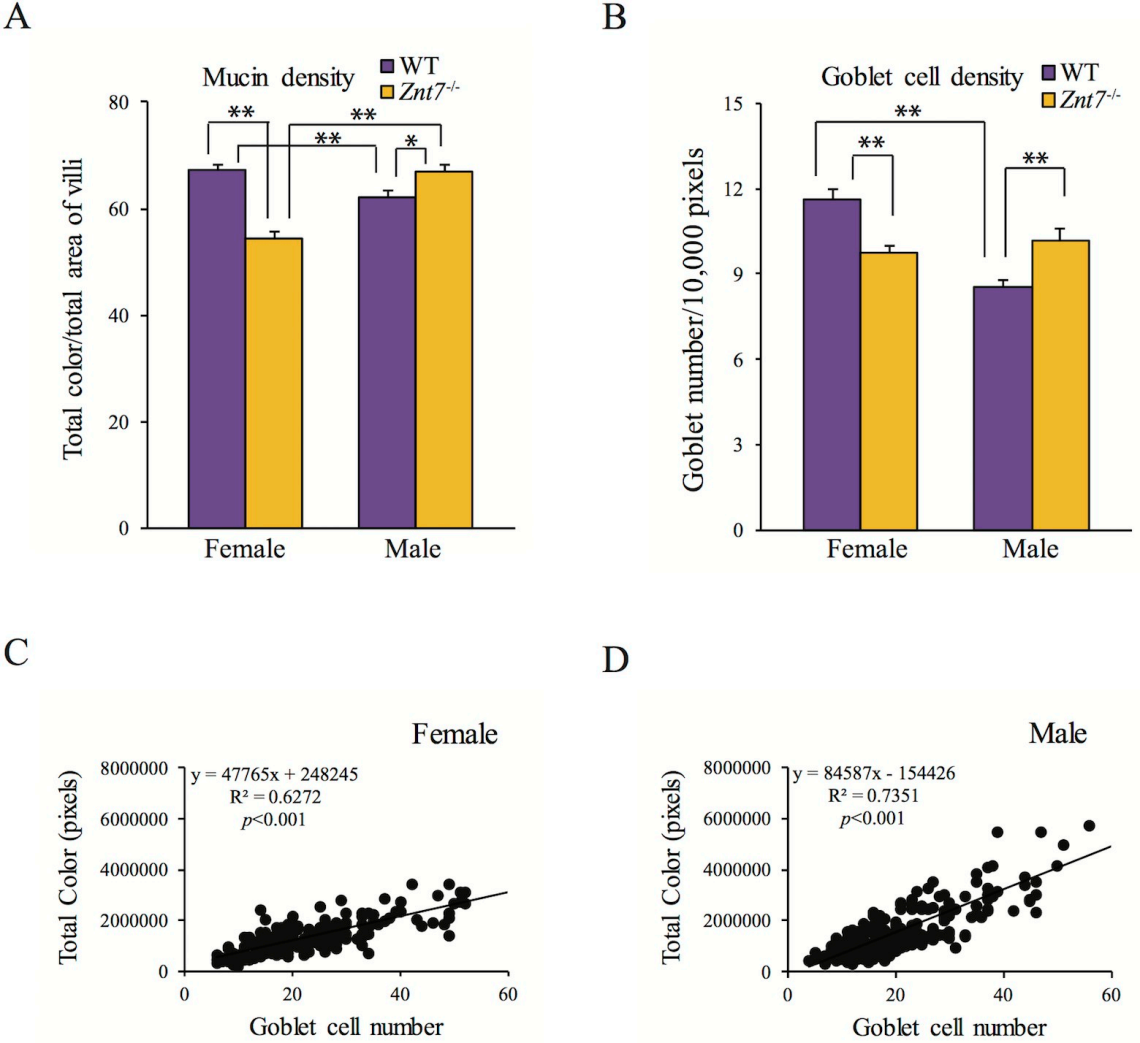
Sex specific differences in mucin density and goblet cell numbers in the colon. (A) Quantification of mucin density measured by Alcian blue/PAS staining in the villi of male and female WT and *Znt7*^*-/-*^ mice. (B) Goblet cell numbers in the villi of the same mice. (C and D) Correlation of mucin density measurement with goblet cell numbers from females and males, respectively. Total color in pixels represents the sum of magenta, blue, and purple intensities, as described in Fig 2, to represent total mucin content. All values are expressed as mean±S.E. (*error bars*), n = 15–18 villi from 5–6 mice/sex/genotype. *, *p*<0.05; **, *p*<0.01.

Compared to WT, the total mucin content in the villus of female *Znt7*^-/-^ mice, including both neutral and acidic mucins, decreased by 18.9±2.4% (*p*<0.01) (Fig 3A). Unlike the female *Znt7*^-/-^ counterpart, male *Znt7*^-/-^ mice showed a very slight increase in the total mucin density per villus compared to WT. Thus, when compared to male *Znt7*^-/-^ mice, the total mucin density in female *Znt7*^-/-^ mice was less (*p*<0.01) (Fig 3A). We next investigated whether the changes in mucin density in the villus of *Znt7*^-/-^ mice was due to the mucin production or the number of goblet cells. As shown in Fig 3B, in female *Znt7*^-/-^ mice, the average number of goblet cells per 10,000-pixel area of the villus was 10±0.3 (5 mice, n = 15 villi), a 15.5% reduction (*p*<0.01) compared to WT (12±0.5, 6 mice, n = 18 villi). On the other hand, the number of goblet cells was increased in male *Znt7*^-/-^ mice relative to WT (*p*<0.05) (Fig 3B). Correlation analyses indicated a positive association between mucin content and goblet cell numbers in both males and females of all genotypes (*p*<0.001) (Fig 3C and 3D), suggesting goblet cell abundance was negatively impacted by the *Znt7* knockout in female mice, but mucin production per goblet cell remained relatively unaffected. This conclusion was further supported by gene expression analysis of *Muc2* (Mucin 2). The *Muc2* gene encodes a secretory and oligomeric mucus gel-forming protein which is a prominent component of the mucus barrier of the colon secreted by goblet cells [22]. Quantitative RT-PCR analysis of *Muc2* in the proximal colon of *Znt7*^-/-^ and WT mice showed a gene expression profile that mirrored the relative number of goblet cells observed (S1 Fig). However, differences in *Muc2* expression did not reach statistical significance. Finally, it should be noted that, in WT mice, the total goblet cell numbers in the proximal colon was significantly higher in females than males while no such sex-dependent difference was observed in *Znt7*^-/-^ mice (Fig 3B).

### *Znt7-knockout* altered expression of only three other zinc transporters

**Fig 4:**
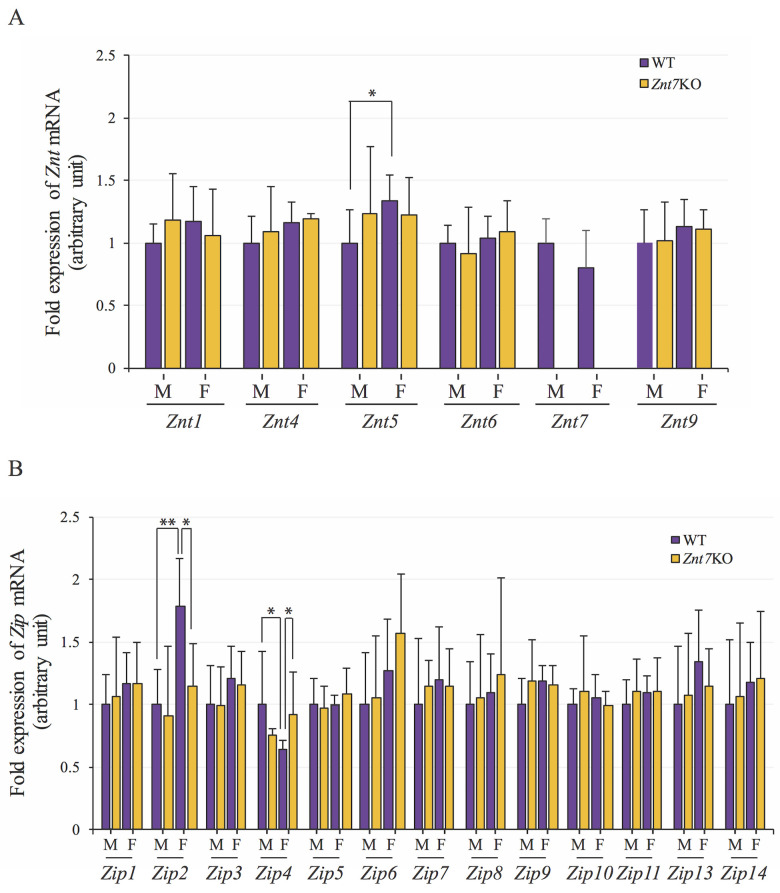
mRNA expression of the *Znt* and *Zip* genes in the mouse colon. (A) mRNA expression of the *Znt* genes *1–10* and (B) mRNA expression of the *Zip* genes *1–14* in colonic tissue distal to the cecum measured by qRT-PCR. The expression of *Actb* was used as an internal reference for quantitation of target gene expression using the 2^–ΔΔCt^ method [23]. Values represent the average of technical triplicates for n = 4–5 mice/genotype/sex. The expression of the target genes was compared to the transcription level in male WT by Student’s *t*-test. *, *p*<0.05; **, *p*<0.01. WT, wild type; *Znt7*KO, *Znt7* knockout.

The null mutation of *Znt7* may have deleterious effects on zinc homeostasis of the epithelium of the colon leading to a compensatory alteration in the abundance of other zinc transporters. We, therefore, analyzed the abundance of mRNA transcripts of other zinc transporters, including the *Slc30a* (*Znt1-10*) and *Slc39a* (*Zip1-14*) family members in the proximal colons from WT and *Znt7*^-/-^ mice. Among 24 zinc transporters examined, we detected little to no mRNA expression for *Znt2*, *Znt3*, *Znt8*, *Znt10*, and *Zip12*. Neither could *Znt7* be detected in the *Znt7*^-/-^ colonic tissue. Among the remaining 19 expressed zinc transporter genes, only three zinc transporter genes, *Znt5*, *Zip2*, and *Zip4*, displayed differential expression between genotypes or sexes (Fig 4). The mRNA expression of *Znt5* appeared to be associated with gender. Female WT mice expressed more *Znt5* (1.3-fold; *p*<0.05) than male WT mice (Fig 4A). This was also true for the expression of *Zip2* with female WT mice having 1.8-fold higher (*p*<0.01) mRNA transcripts than male WT mice. On the other hand, the mRNA expression of *Zip4* was less (40%; *p*<0.05) in female WT mice than the male counterpart in the colon (Fig 4B). Interestingly, the impact of the *Znt7*-null mutation on the gene expression of zinc transporters in the proximal colon seemed limited. We found that only *Zip2* and *Zip4* gene expression was affected by the *Znt7*-KO (Fig 4B) and, remarkably, these changes only happened in females with *Znt7*^-/-^ mice expressing ~36% less *Zip2* (*p*<0.05) and ~44% more *Zip4* (*p*<0.05) relative to WT. It is worth noting that the changes of *Zip2* and *Zip4* gene expression in response to the loss of *Znt7* in the female colon was in opposite direction of the expression observed in WT females compared to WT males.

### *Znt7* knockout showed a sex specific effect on the gut microbiome composition

MiSeq sequencing of the bacterial 16S rRNA V4 region from 10-week old mouse stool pellets yielded a total of 4,437,644 sequences with an average of 85,339 sequences per sample. Trimming, quality filtering and deconvolution of the sequencing data using DADA2 in the QIIME2 software platform yielded 527 total unique bacterial features (sequence variants) representing 53 taxa identifiable to the order, family or genus level.

**Fig 5:**
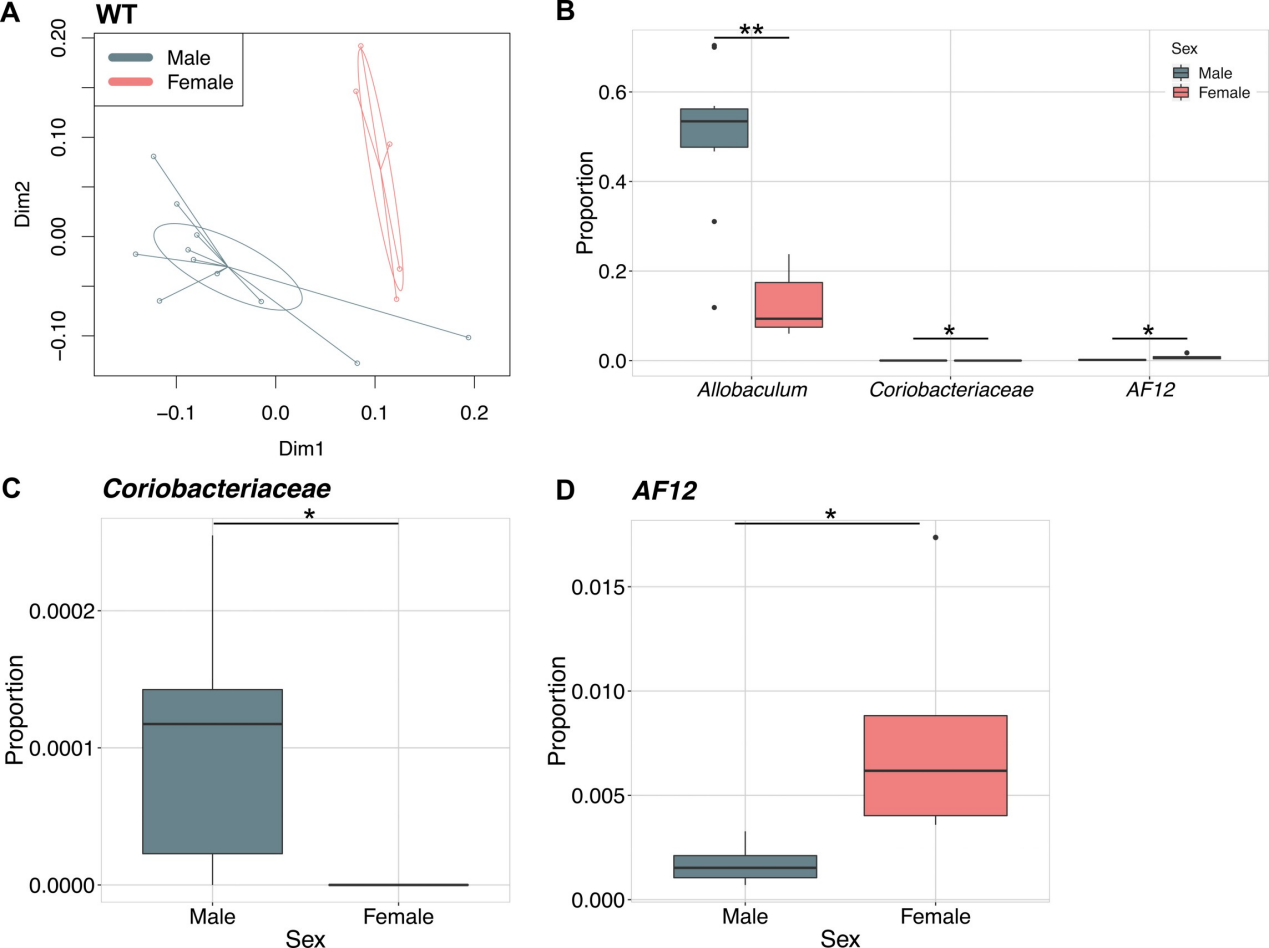
Microbial community structure differs between male and female *Znt7*^*+/+*^(WT) mice. (A) Principal coordinate analysis of the weighted UniFrac distance measures between gut microbial communities from male and female WT mice. (B) Boxplots of the relative abundances of bacterial taxa that were significantly differentially abundant between male and female mice as determined by DESeq2 analysis. (C and D) Boxplots of the relative abundance of (C) *Coriobacteriaceae* and (D) *AF12* with the y-axes adjusted showed differences between sexes more clearly than in panel B. *, *p*<0.05; **, *p*<0.01.

Because sex differences were observed in the mucin density of these mice, we first examined overall microbial community composition (beta diversity or between sample diversity) of the gut microbiome between WT male and WT female mice using principal coordinates analysis of weighted UniFrac distances. Male and female gut microbial community compositions differed significantly (adonis *p* = 0.002, Fig 5A). These overall differences were driven primarily by a difference in the relative abundance of *Allobaculum* (from phylum *Firmicutes*), which was more abundant in male than in female mice (DESeq2 *p* = 0.000118, Fig 5B). Additionally, *Coriobacteriaceae* (from phylum *Actinobacteria*), though much less abundant in general, was present in male mice, but undetectable in female mice (DESeq2 *p* = 0.0104, Fig 5B and 5C). Finally, there was significantly more *AF12* (family *Rikenellaceae* in phylum *Bacteroidetes*) in WT female than male mice (DESeq2 *p* = 0.0173, Fig 5B and 5D). Therefore, the effect of *Znt7* knockout on the gut microbiome composition was explored further in a sex specific manner.

**Fig 6:**
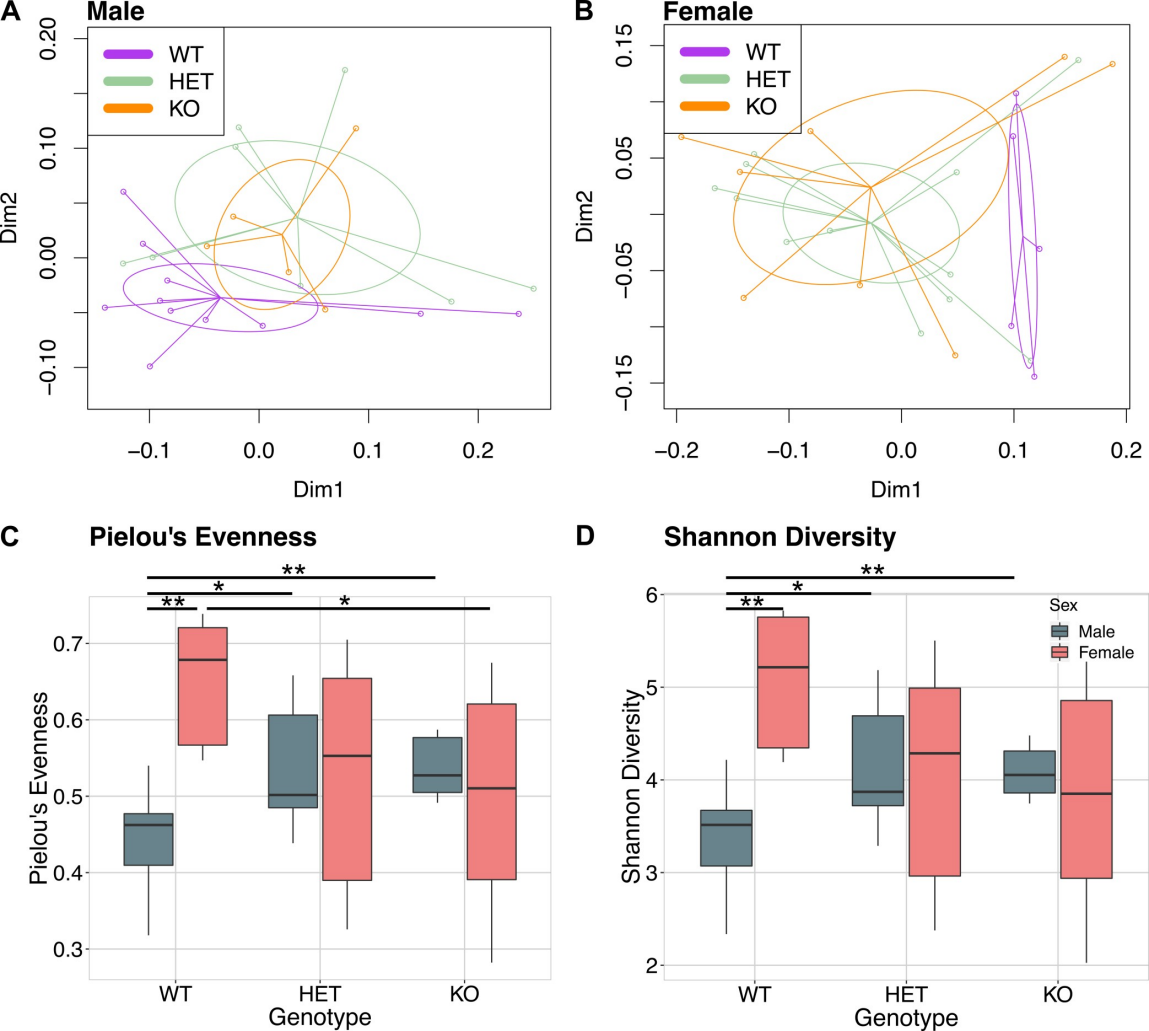
Sex specific effects of *Znt7* knockout on overall bacterial community structure. (A and B) Principal coordinate analysis of the weighted UniFrac distance measures between microbial communities from *Znt7*^*+/+*^ (WT), *Znt7*^*+/-*^ (HET) and *Znt7*^*-/-*^ (KO) in (A) male and (B) female mice. (C and D) Boxplots of microbial community evenness, represented by Pielou's evenness index and Shannon diversity index respectively. *, *p*<0.05; **, *p*<0.01.

Principal coordinates analysis of weighted UniFrac distances between samples from the three genotypes showed a visual clustering of WT mice away from *Znt7*^+/-^ and *Znt7*^-/-^ in both male and female mice that was not significant by permutational ANOVA (adonis *p* = 0.188 and *p* = 0.15, respectively) (Fig 6A and 6B). Out of four measures of alpha diversity used to determine potential differences in within-sample diversity, a significant interaction effect of sex and genotype was found for only Pielou’s evenness (two-way ANOVA, *p* = 0.008942) (Fig 6C) and Shannon diversity (two-way ANOVA, *p* = 0.00954) (Fig 5D and S2 Fig). Pairwise differences in both evenness and Shannon diversity were significant between sexes in WT mice (Welch’s *t*-test, evenness *p* = 0.002955, Shannon *p* = 0.003988), but not in the *Znt7*^+/-^ or *Znt7*^-/-^ genotype (evenness *Znt7*^+/-^ *p* = 0.942, *Znt7*^-/-^ *p* = 0.5437; Shannon *Znt7*^+/-^ *p* = 0.9151, *Znt7*^-/-^ *p* = 0.6066) (Fig 6C and 6D). In male *Znt7*^+/-^ and *Znt7*^-/-^ mice, microbial communities were significantly more even than the WT controls (Welch’s *t*-test, evenness *Znt7*^+/-^ *p* = 0.01922 and *Znt7*^-/-^ *p* = 0.005522; Shannon *Znt7*^+/-^ *p* = 0.01817, *Znt7*^-/-^ *p* = 0.005289). The trend was opposite in female mice, with *Znt7*^-/-^ communities showing significantly less evenness than the WT control (Welch’s *t*-test, evenness *p* = 0.04555; Shannon *p* = 0.05124) (Fig 6C and 6D).

**Fig 7:**
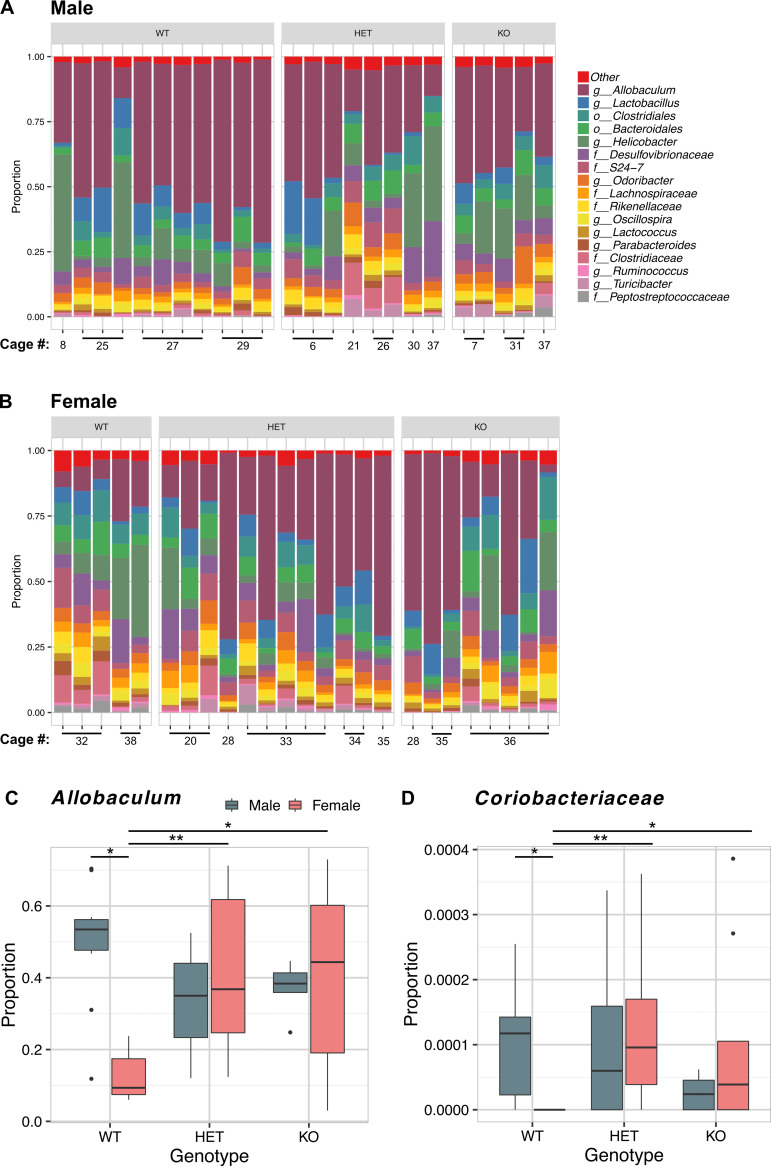
Bacterial taxa significantly differed by *Znt7* genotype in a sex dependent manner. (A and B) Barplots showing proportions of bacterial taxa present in mouse feces. The microbial community composition of each mouse is represented by a single bar. The proportions of taxa are shown on the y-axis and the cage that each mouse was housed in during the study is shown on the x-axis. Taxa present at less than 2% relative abundance were grouped into the “Other” category. The most specific taxonomic classification obtained is shown and the displayed taxon level is represented by a single letter code preceding the classification; o = order, f = family, g = genus. (C and D) Boxplots of the relative abundance of *Allobaculum* and *Coriobacteriaceae* respectively within WT, *Znt7*^+/-^ (HET) and *Znt7*^-/-^ (KO) mice. A log ratio test, followed by pairwise testing performed in DESeq2 showed a significant sex-specific effect of genotype for these bacterial taxa. *, *p*<0.05; **, *p*<0.01.

*Allobaculum*, *Helicobacter*, *Lactobacillus* and unidentified members of the orders *Clostridiales and Bacteriodales* were the five most abundant bacterial taxa detected (Fig 7A and 7B). Evenness was negatively correlated with the proportion of *Allobaculum* (Spearman rho = -0.8667347, *p*<2.2 x 10^−16^), and positively correlated with the abundance of *Bacteroidales* and *Clostridiales* (Fig 7 and S3 Fig). No significant relationship to *Helicobacter* or *Lactobacillus* was detected (S3 Fig).

DESeq analysis showed that *Allobaculum* was differentially abundant among genotypes in a sex dependent manner (DESeq log ratio test, *p* = 0.01565, Fig 7C). Subsequent pairwise comparisons among the experimental groups showed that *Allobaculum* was significantly more abundant in female *Znt7*^+/-^and *Znt7*^-/-^ (DESeq Wald *t*-test, *p* = 0.00444 and *p* = 0.0120, respectively) than in female WT mice (Fig 7C). Significant differences in the proportion of the less abundant unidentified members of the family *Coriobacteriaceae* were also detected (DESeq log ratio test, *p* = 0.00129) (Fig 7D). Similar to *Allobaculum*, the *Coriobacteriaceae* family was significantly more abundant in female *Znt7*^+/-^and *Znt7*^-/-^mice than in female WT mice (DESeq Wald *t*-test, *p* = 0.00118 and *p* = 0.0308, respectively). In both cases, the differences in abundance of specific bacterial taxa between sexes in WT mice were reduced with *Znt7* knockout (heterozygous or homozygous) as there were no significant differences in abundance of these taxa between sexes of *Znt7*^+/-^or *Znt7*^-/-^mice (Fig 7C and 7D).

The experimental groups were separated by cage as shown in Figs 6A and 7B. Bacterial sequence counts rarefied to 36,481sequences per sample and averaged by cage, showed the same trend in relative abundance of *Allobaculum* between experimental groups as described for the evaluation of individual mice (S4 Fig). However, the trend did not reach statistical significance (DESeq log ratio test, uncorrected *p* = 0.0061, FDR corrected *p* = 0.15).

### Microbial community composition is weakly correlated with mucin density

**Fig 8:**
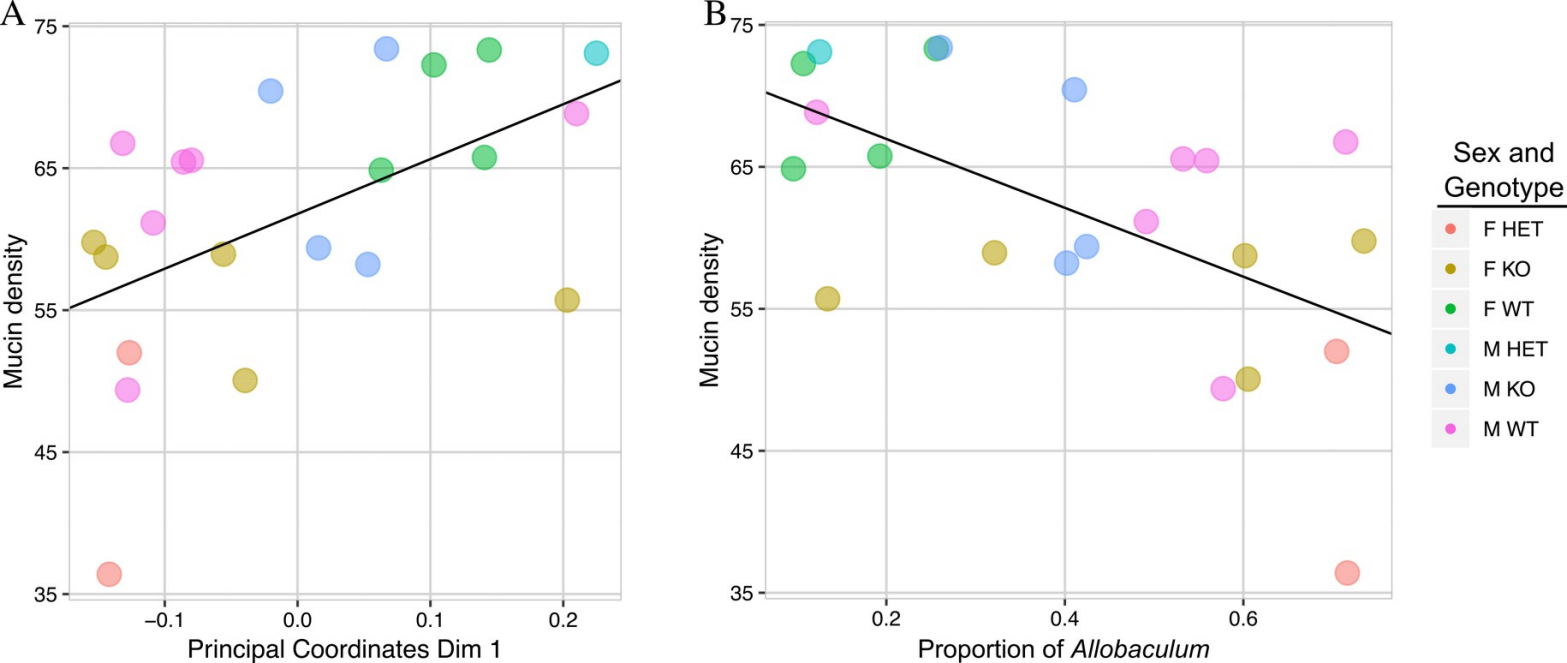
Relationship between mucin density and microbial community in the mouse colon. (A and B) The y-axes in each graph show mucin density values for a subset of the mice. The x-axis shows (A) the first dimension of principal coordinates from the analysis of weighted UniFrac measurements between the microbial communities or (B) the relative proportion of *Allobaculum* in each 10-week old mouse. A regression line describing the linear relationship between each of the two variables and mucin density is shown. Spearman rho = 0.53472, *p* = 0.01142 and Spearman rho = -0.53811, *p* = 0.01083 for each of the comparisons respectively.

We next examined whether mucin density and microbiome composition were correlated. The first dimension of the principal coordinates analysis of weighted UniFrac distance measures among microbial communities were used to represent the overall microbial community composition for which mucin density was examined in all experimental groups at 10 weeks of age. This showed a moderate, but significant (Spearman rho = 0.53472, *p* = 0.01142) correlation with mucin density (Fig 8A). Because the change in the relative abundance of *Allobaculum* was the most profound across the experimental groups, we next examined the relationship between *Allobaculum* and mucin density. The relative abundance of *Allobaculum* was weakly and negatively correlated (Spearman rho = -0.53811, *p* = 0.01083) with the total mucin density measured in these mice (Fig 8B).

## Discussion

Examination of the relationship between zinc homeostasis, intestinal mucosal composition and gut microbiome composition is important for understanding the role of zinc in human health. We hypothesized that systemic zinc deficiency, previously observed in *Znt7*-KO mice, was due to altered cellular zinc homeostasis in goblet cells in the gut, leading to reduced mucin production and altered microbial composition. To investigate this hypothesis, we fed a semi-purified diet containing 30 mg Zn/kg to *Znt7*^*-/-*^ mice with their heterozygous and wild type littermates and found a sex specific effect on colonic mucin density, goblet cell number, and microbiome composition. Intestinal composition was more dramatically affected in *Znt7*^*-/-*^ female mice, causing them to have values that were more equivalent to males.

The effects of *Znt7* knockout on systemic zinc status and body fat accumulation in the mouse colony used in these experiments have previously been characterized [9, 10]. We confirmed that *Znt7* knockout has been maintained in this colony and also examined the expression of other *Znt* and *Zip* genes. Importantly, the majority of the other zinc transporter genes expressed in the colon were unchanged. Only *Zip2* and *Zip4* were significantly affected by the *Znt7* knockout. Little is known about the function and expression profile of *Zip2* [24]. Therefore, the significance of the differential expression of this gene in female *Znt7*^*-/-*^ mice is unknown. However, like *Znt7*, *Zip4* is highly expressed in the colon and is involved in absorption of dietary zinc [25–27]. Therefore, it is likely that the increase in expression of *Zip4* in female *Znt7*^*-/-*^ mice relative to WT is a compensatory change in response to the decreased absorption of dietary zinc occurring in female *Znt7*^*-/-*^ mice. Interestingly, *Zip4* was not significantly affected in male mice, possibly because expression was much greater in WT males to begin with. Furthermore, it is known from previous studies that this compensatory increase in *Zip4* expression is not likely sufficient to alleviate zinc deficiency in *Znt7*^*-/-*^ mice [9, 10].

Female *Znt7*^*-/-*^ mice showed reduced mucin and goblet cell density relative to their wild type counterparts, while male *Znt7*^*-/-*^ mice showed an opposing increase. It has previously been described that the colonic mucin thickness is greater in female mice than in male mice [28], which is supported by the observations in WT mice from the current study. We hypothesized that potential disruption of zinc homeostasis in the goblet cells would affect mucin production in the colon. In female *Znt7*^-/-^ mice, the observation of decreased mucin density and decreased goblet cell numbers relative to WT supported this hypothesis. However, the increase in total numbers of goblet cells in male *Znt7*^-/-^ mice compared to the control, suggests sex may affect the relationship between zinc homeostasis and goblet cell population in the colon. Indeed, it has been shown that female sex hormones play a significant role in promoting gut mucosal health and mucosa associated immune function by the estrogen-dependent signaling pathway [29–31]. In humans, pre-menopausal females display more robust humoral and immune function than their male counterparts [32] and it is well known that zinc is critical for immune function systemically and within the gut [33, 34]. Therefore, it is possible that the gender variation in mucin density and goblet cell population observed in this study occurs through regulation of the innate immune response in the colonic epithelium [35, 36]. It is likely that cellular zinc deficiency induced by *Znt7* knockout has more profound impact on the goblet cell replenishing and total mucin production in females than males. However, this possibility would have to be explored further in future studies.

Mucin composition can drive the diversity of the gut microbiome within the colon and vice versa [37, 38]. The relative abundance of *Allobaculum* was different between sexes in WT mice and was increased in female mice with *Znt7* knockout, but was not significantly altered in male mice. Mice are coprophagic and it is therefore possible that because mice were housed and separated by the genotype, our results might be confounded by cage effects. However, taking the average microbial community of mice per cage and performing the same analysis showed the same trend in relative abundance of *Allobaculum*. Additionally, we found a negative correlation between mucin density and the relative abundance of *Allobaculum* in the mouse colon. The type strain of this genus, *Allobaculum stercoricanis*, is a strictly anaerobic, rod shaped bacterium that was originally isolated from dog feces [39]. It has the ability to ferment glucose and disaccharides (cellobiose, fructose, galactose, glucose, maltose, and sucrose) and to produce lactate and butyrate during fermentation [39], suggesting that it has the potential to benefit the health of the colonic epithelium.

Previous studies have shown that a high fat diet in mice was associated with decreased relative abundance of *Allobaculum* [40–42]. Ravussin et. al. found that the abundance of *Allobaculum* was decreased with administration of a high fat diet and further that *Allobaculum* was positively correlated with weight reduction in mice [41]. Everard et. al. and Van Hul et. al. also showed that the relative abundance of *Allobaculum* was decreased by a high fat diet in mice and increased with the addition of the prebiotic oligofructose to the mouse drinking water [43] or grape pomace extract to the mouse feed [42]. Together, these earlier results suggest that the carbohydrates within the colon may play a role in the abundance of *Allobaculum* within the mouse gut.

The amount of zinc present in the research diets used for two of the above studies was not dramatically different between the high fat and control diets (Ravussin et. al.: 37.7 mg Zn/kg diet in high fat diet, 27.7 mg Zn/kg diet in control [41]; Everard et. al.: 37.7 mg Zn/kg diet in high fat diet, 55 mg Zn/kg diet in control diet [43]). Additionally, we observed a difference in the relative abundance of *Allobaculum* between female WT and *Znt7*^*-/-*^ mice despite the fact that the diet was the same for all of the mice in the study described here. Therefore, we predict that differences in composition of the mucin by sex and *Znt7* status may affect carbohydrate availability in the colon. For example, mucin degrading gut bacteria can process mucin into smaller saccharide subunits, including galactose [44], which could then be utilized by *Allobaculum* as a carbon source. Decreased mucin production might therefore reduce this food source for *Allobaculum* leading to the negative association between mucin contents and abundance of *Allobaculum* that we observed. However, further studies are needed to confirm a causal relationship.

Taken together, our findings show that *Znt7* knockout affects goblet cell number and mucin thickness in mice, which is linearly correlated with gut microbial community composition. This linear relationship, which was agnostic to sex, suggests that the primary driving force in altered gut microbial composition with *Znt7* knockout is the change in mucin composition. The novel finding that the relationship between zinc uptake and mucin thickness is sex dependent opens a new field of study and suggests that zinc regulated functions within the colon may be different between sexes.

## Conclusions

*Znt7* knockout leads to reduced mucin density in female, but not male mice. Changes in the relative abundance of *Allobaculum* were also uniquely significant in female mice. The gut microbial composition and relative abundance of *Allobaculum* both correlated with mucin density across sex and genotype, suggesting that sex dependent effects of *Znt7* knockout on mucin density lead to changes in the gut microbial community. Additional studies are needed to confirm a causal relationship between *Znt7*, mucin production and growth of *Allobaculum*.

## Materials and methods

### Animals and diets

All animal experiments were performed in strict accordance with the recommendations in the Guide for the Care and Use of Laboratory Animals of the National Institutes of Health and the American Veterinary Medical Association and with approval from the Animal Care Committee of the University of California at Davis.

*Znt7*^-/-^ mice with a congenic C57BL/6J (B6) genetic background were maintained from the line that was generated and described previously [45]. The homozygosity of *Znt7*-KO was maintained by heterozygous x heterozygous breeding. The experimental animals, *Znt7*^+/+^, *Znt7*^+/-^ and *Znt7*^-/-^ used in this study were obtained by heterozygous breeding. Mice were weaned at 3 weeks old and fed a standard laboratory chow diet (Laboratory Rodent Diet 5001, LabDiet, Brentwood, MO, USA; the zinc content of the diet was 79 mg Zn/kg diet). When mice reached 6-week old, they were fed a semi-purified diet containing 30 mg Zn/kg *ad libitum* (Research Diets, New Brunswick, NJ, USA) until 10 weeks of age. Body weights were recorded every week after mice were on the special diet. The experimental mice were housed between 2-5/cage, separated by genotype and sex. All mice were housed in a temperature-controlled room at 22–24°C with a 12 h light:dark cycle. Mice were euthanized at 10 weeks of age by cardiac puncture under general anesthesia (intraperitoneal injection of 100 mg/Kg ketamine and 10 mg/Kg xylazine, MWI Veterinary Supply, Boise, ID, USA). Euthanasia was confirmed by cervical dislocation. The colon was collected at the necropsy and further processed for mucin staining. All animal experiments were conducted in accordance with National Institutes of Health Guidelines for the Care and Use of Experimental Animals and were approved by the Institutional Animal Care and Use Committee of the University of California Davis.

### Tissue dissection, fixation, process, and embedding

The colon was carefully dissected and the colonic tissue 1 cm distal to the cecum was fixed in pre-chilled Carnoy’s solution containing 60% ethanol, 30% acetic acid, and 10% chloroform for 2 ~ 4 h on ice. The tissue was then rinsed under running distilled water to remove excess Carnoy’s solution, placed in 70% FLEX (Richard-Allan Scientific, San Diego CA, USA) and stored at 4°C until ready to process. Before processing, the colon tissue was placed in a cassette. The cassette was then put in a beaker containing 80% FLEX and incubated at room temperature for 30–60 min. The tissue was processed in a STP 120 tissue processor (ThermoFisher Scientific, Carlsbad, CA, USA) with a program set up as follows: 80% FLEX for 30 min once, 100% FLEX for 30 min twice, clear rite for 30 min twice, and paraffin type 9 wax (Richard-Allan Scientific) for 20 min twice. Cassettes were removed from the processor and put into a beaker containing paraffin type 9 wax. Wax was degassed for 10 min in a vacuum oven (VWR Scientific Inc., Bridgeport, NJ, USA) with the vacuum pressure set at 17.5 inHg and temperature at 60°C. The degas procedure was repeated once followed by embedding using a HistoStar embedding equipment (ThermoFisher Scientific).

### Mucin staining of the colon

The colon tissue was cut into 5-μm sections and placed on positively charged slides. Sections were rehydrated as follows: xylene (ThermoFisher Scientific) for 3 times, 100% FLEX (ThermoFisher Scientific) twice, 95% FLEX once, 70% FLEX once, 50% FLEX once with 3 min for each step. Mucins were stained as described previously [46]. Periodic acid Schiff’s reagent and Alcian blue were used to distinguish neutral (bright red magenta) and acidic (dark blue) mucins. Nuclear Fast Red was used to stain the cell nucleus (red) for better visualization of the tissue under a light microscope. Briefly, sections were stained in 0.05% Alcian blue (ThermoFisher Scientific), made in 3% acetic acid, for 15 min and then rinsed in running tap water (3 min) followed by distilled water (2 min). Sections were then stained with 0.05% periodic acid for 5 min, washed in running tap water for 3 min, and then rinsed in distilled water for 5 min. Next, sections were stained with the Schiff’s reagent (Ricca Chemical Company, Arlington, TX, USA) for 10 min, washed in running tap water for 3 min and then distilled water for 5 min. Finally, sections were stained with Nuclear Fast Red for 5 min followed by washing the sections in running tap water 3 min and in distilled water for 5 min. The sections were then allowed to dry and a cover slip was applied using Permount^®^ mounting medium (ThermoFisher Scientific).

### Microscopic examination of the colon

A Nikon E800 microscope equipped with a Diagnostic Instruments SPOT RT camera and SPOT 5.0 image acquisition software was used for acquiring images (Diagnostic Instruments Inc., Sterling Heights, MI, USA). Each image was taken using the same manual settings and white balance in three areas of the proximal colon per mouse were examined and three to six micrographs per slide (20x magnification) were taken.

### Quantification of mucins in the goblet cells of the colon

TissueQuant software [21] was used for color intensity quantification of the colon. Images were first processed using Photoshop software (Adobe, San Jose, CA, USA) to crop villi for quantification. For each image, six villi from the left to right of each image were circled and saved separately as new image files for TissueQuant analysis. Within the TissueQuant software, quantification parameters were set to recognize deep blue, dark blue/purple, and magenta. The three colors were then quantified as the product of value and area, and the color density (mucin density) of each villus was equal to the sum of three colors divided by the total villus area. The goblet cell numbers per villus were counted. The mucus density was calculated by dividing the sum of the total mucin color by the total villus area. Mucin content per goblet cell was calculated by dividing the sum of the total mucins by the total numbers of goblet cells. The goblet cell density in the villus was calculated by dividing the numbers of goblet cells by the total villus area.

### Feces collection and bacterial DNA extraction

Fresh stools were collected from the experimental mice at 08:30–10:00 four weeks after the introduction of the special diet. Briefly, mice were held at the scruff with the base of the tail exposed. This holding position made mice producing feces quickly. Freshly expelled fecal pellet was then captured at the anus with a sterilized forceps and placed in a RNase- and DNase-free 1.5 mL Eppendorf tube. Samples were immediately frozen on dry ice after collection and subsequently stored at 80°C until use. Bacterial DNA was isolated from fecal pellets using a Nucleospin Soil Kit (Macherey-Nagel GmBH & Co., Düren, Germany) according to the manufacturer’s instructions with some modifications. Briefly, approximately 200 mg of fecal sample was lysed in 700 μL lysis buffer SL2 with 150 μL enhancer SX in a Nucleo-Spin®Bead Tube Type A and kept on ice for 5 min. The samples were then vertically shaken using a Geno/Grinder® 2010 (SPEX®SamplePrep, Metuchen, NJ, USA) at 1,750 strokes/min for 2 min and placed on ice for 1 min. The shaking procedure was repeated twice. After homogenization and cell disruption, the samples were chilled on ice for 1 min followed by centrifugation at 11,000 g for 1 min. The subsequent procedures for DNA purification were carried out based on the manufacturer’s instructions. The purified DNA were quantified using a NanoPhotometer® P300 (Implen Inc., Westlake Village, CA, USA) and stored at -20°C until use.

### Total RNA isolation, cDNA synthesis, and quantitative PCR

Frozen colonic tissue (~1 cm long) right after the cecum was crushed on dry ice and approximately ½ of the amount was homogenized in a Dounce Tissue Grinder with a tight pestle (Wheaton, Millville, NJ) in the presence of 1 mL of the TRIzol^®^ reagent (ThermoFisher Scientific) according to the manufacturer’s instructions. Total RNA (500 ng) was converted to cDNA using a High Capacity cDNA Reverse Transcription Kit (ThermoFisher Scientific) following the manufacturer’s protocol. cDNA was then diluted 1:5 or 1:10 and quantitative PCR reactions were performed in using a PowerUp™ SYBR™ Green Master Mix (ThermoFisher Scientific). The sequences for primer sets used in the quantitative PCR are provided in Table 1. Fold difference of the target gene expression was calculated using the 2^–ΔΔCt^ method [23]. The expression of *Actb* was used as a housekeeping gene for quantitation of the expression of target genes.

**Table 1:** Primer sequences.

Gene Symbol	Forward primer	Reverse primer	Reference
*Muc2*	CTGACCAAGAGCGAACACAA	CATGACTGGAAGCAACTGGA	Zarepour et al., 2013
*Muc2*	GCTGACGAGTGGTTGGTGAATG	GATGAGGTGGCAGACAGGAGAC	Wlodarska et al., 2011
*Slc30a1*	AGGAGGAGACCAACACGCT	TGTACATCCACTGGGTCATCA	Tepaamorndech et al., 2016
*Slc30a2*	TGCACGGCCAGGTACACACGA	GGCCCGACGCAAACTCTATGT	Tepaamorndech et al., 2016
*Slc30a3*	CCTAGTGAGTGCCCGAGACC	CAAGCTTGGGCTCCTCAG	Tepaamorndech et al., 2016
*Slc30a4*	CTGCCGTCCTCTACTTGCTT	GCATATGGAGTGCATCTGTCA	Tepaamorndech et al., 2016
*Slc30a5*	CCACCTGACCATGAAAAAGAA	CAATACTGGCAGAATGGCG	Tepaamorndech et al., 2016
*Slc30a6*	AGCTGTACGGCTCCTTACCAT	GGTCAGCTGCGACAAGTCTA	Tepaamorndech et al., 2016
*Slc30a7*	GCAGATCCTATCTGTTCGATTCT	CCAACGAGGGAGGAGTTCTT	This study
*Slc30a8*	TGCCAAGTGGAGACTCTGTG	AGCCGCATCAGTGAGGATAG	Tepaamorndech et al., 2016
*Slc30a9*	GGCTCACAGACTCCAAAAGC	AGGGTCTTGCTTAAGTGGAGC	Tepaamorndech et al., 2016
*Slc30a10*	CACTTCTGGAGCTTCTATGCTTCGGT	GGAATACCAGGCTGCCAGCAGAAA	Tepaamorndech et al., 2016
*Slc39a1*	CATAGATGAGGCCTTGGAGG	ATCTGCTCCATCACCAGGAC	This study
*Slc39a2*	GCCGCTGGCACGTTTTTAT	CAGCAGCCACACAGCTATAC	This study
*Slc39a3*	CTTTAATGCACTGCTGCCTG	CACCATCATGAGCGTCTCC	This study
*Slc39a4*	AGAAGATTGAGGCCCATGC	CTTTTGGAAACCCCTGCTG	This study
*Slc39a5*	TGGCTGACCATCTGAATGAG	GAACTGACGAGGGGTCAGAG	This study
*Slc39a6*	TGTGGAACACGTACTCACACTG	ACAGCTGCTTCTTGCTCTCC	This study
*Slc39a7*	AGTCCTGCTGCACGAACTG	CAATTGCAGTCACGAGTTGC	This study
*Slc39a8*	TGTGACTTGCTATGCCAACC	TTGGCTCTGTTTTTCCATCC	This study
*Slc39a9*	CCTTTCAGAGGTCAATGCCAC	CACTTCAGGGAGGACATG	This study
*Slc39a10*	AGATGCACACGGCACTCG	GTGGTTGCAATGATGGAAAA	This study
*Slc39a11*	GACCCTGCATTGATGAAGAA	TATACCTCGCCATTCTCACG	This study
*Slc39a12*	CAGTGACTGCTGGGATGTTC	CAGGAGAAACGTCATCCAGG	This study
*Slc39a13*	GTTTTCCCCTTGCTGGTCAT	GTCCACCTAAGGCAAAGCTG	This study
*Slc39a14*	CCCTGGATAGTGAGGCTGC	CTGGTCAAACTGAGCCAACA	This study
*Actb*	TCATGAAGTGTGACGTTGACATCCGT	CCTAGAAGCATTTGCGGTGCACGATG	Huang et al., 2012

### PCR and PCR product purification

The V4 hypervariable region of the 16S ribosomal RNA (rRNA) gene from fecal bacteria DNA samples (40 ng) was amplified using primers F515 (5’- GTGTGCCAGCMGCCGCGGTAA-3’) and R806 (5’- GGACTACHVGGGTWTCTAAT-3’) [47] with Takara Ex Taq DNA polymerase (Takara Bio Inc. Kusatsu, Shiga Prefecture, Japan). A unique eight nucleotide barcode on the 5’-end of the forward primer was used to amplify each sample [48, 49]. Double distilled water and *E*. *coli* DNA were used as a negative and a positive control, respectively. PCR was performed at 94°C for 3 min for 1 cycle followed by 25 cycles of 94°C for 45”, 55°C for 30”, and 72°C for 30” and 1 cycle of 72°C for 10 min. The PCR products were then pooled and subjected to gel-purification using a QIAquick Gel Extraction kit (Qiagen Inc., Valencia, CA, USA) according to the manufacturer’s instructions.

### 16S sequencing and data analysis

Library preparation and sequencing on Illumina MiSeq were performed at the UC Davis DNA technologies core (https://dnatech.genomecenter.ucdavis.edu/). FASTQ files containing 16S rRNA gene sequences were analyzed using the open source software QIIME2 version 2018.11 (https://qiime2.org/) [50]. The files were imported using the file type classification “MultiplexedSingleEndBarcodeInSequence”. The cutadapt plugin [51] was used to de-multiplexed sequences using the barcode occurring within the first eight nucleotides of each sequence with no errors allowed. Subsequently, primer sequences were trimmed using a search for the forward primer sequence text, allowing a 20% error rate and up to two occurrences within the 5’ region of the sequence, also using the cutadapt plugin. Additional trimming (to a total length of 220 nucleotides), quality filtering, chimera removal and identification of unique sequence variants was performed using the DADA2 plugin [52]. The quality filtered representative sequences were tabulated in a sequence variant (SV) table. Next the sequences were aligned with the align-to-tree-mafft-fasttree pipeline in the phylogeny plugin, which creates a MAFFT alignment (https://mafft.cbrc.jp/alignment/software/), and a phylogenetic tree, rooted at the midpoint. The rooted tree and SV table were used in calculation of diversity metrics including similarities within sample (alpha diversity) and between sample (beta diversity) using the core-metrics-phylogenetic function within the QIIME 2 diversity plugin. Taxonomy was assigned to each representative sequence a Naïve Bayes pre-trained classifier (250 base pair length sequences in the GreenGenes 13_8 at 99% identity) implemented in the QIIME2 feature-classifier plugin.

### Statistical analysis

Statistical analyses were performed using Student *t*-test for body weights (expressed as means±S.E) and Mann-Whitney u test for the mucin intensity study (data presented as mean±SD). Differences were considered significant at *p*<0.05. The phyloseq [53], vegan (https://rdrr.io/cran/vegan/), ggplot2 [54] and DESeq2 [55] packages, in addition to base functions (kruskal.test and cor.test) in the R statistical software platform were used for analysis of 16S rRNA sequencing data. Samples containing less than 30 sequences, and bacterial features (sequence variants) with less than 10 sequences were removed from the 16S rRNA tabulated data set prior to performing any statistical analysis. Differential abundances of bacterial taxa between experimental groups were measured in the filtered data set using the DESeq2 log ratio test with sex, genotype and the interaction between sex and genotype as the complex model and the individual variables of sex and genotype as the reduced model. Pairwise comparisons (male vs. female and within sex genotype comparisons) of bacterial taxa were performed in DESeq2 with the Wald test, using the single variable of interest on the relevant subset of the data. Pairwise results are only shown for bacterial taxa that were significant using the log ratio test on the full data set. Beta diversity was determined by principal coordinate analysis of weighted UniFrac [56] distances between samples within the QIIME 2 software package. Significant differences in the overall composition of bacterial communities between groups were determined using the adonis function in the vegan package in R. Alpha diversity was determined by faith’s phylogenetic diversity, Shannon diversity, total observed sequence variants and Pielou's evenness within the QIIME 2 software platform. Alpha diversity values were tested for normality using the Shapiro-Wilk test (shapiro.test function in the stats package in R). Those measures that were not normally distributed (Faith’s phylogenetic diversity) were rank transformed using the rank function in base R. A parametric two-way ANOVA (AOV function in the stats package in R) was then used to test for effects of sex, genotype and the interaction between sex and genotype on differences in values of alpha diversity measures.

## Supporting information

S1 Fig mRNA expression of the *Muc2* gene in colonic tissue distal to the cecum. *Actb* was used as an internal reference for calculation of *Muc2* fold difference using 2^–ΔΔCt^ [23]. Values represent the average of technical triplicates for n = 4–5 mice/genotype/sex. Student’s *t*-test was used to compare values to the transcription level in male WT mice. No significant differences were found.
(PDF)

S2 Fig Alpha diversity of mouse fecal microbial communities summarized by sex and genotype. Boxplots of the alpha diversity measured by (A) total observed sequence variants (SVs) or (B) Faith’s Phylogenetic Diversity are shown. There were no statistically significant differences between experimental groups for these measures.
(PDF)

S3 Fig Relationship between evenness and the relative proportion of abundant bacterial taxa within the mouse colon. The alpha diversity measurement of bacterial community evenness is shown (x-axes) relative to the proportions of (A) *Allobaculum*, rho = -0.8667347, *p*<2.2 x 10^−16^, (B) *Bacteroidales*, rho = 0.5289796, *p*<0.001, and (C) *Clostridiales*, rho = 0.5872449, p < 0.0001, (D) *Lactobacillus*, rho = -0.1709184, p = 0.2395 and (E) *Helicobacter*, rho = 0.1867347, p = 0.1983 are shown on the y-axis. A regression line showing the linear relationship between evenness and proportion of each bacterial taxa (Spearman) is shown on each graph.
(PDF)

S4 Fig Microbial communities averaged by cage show the same trends in *Allobaculum* abundance as individual mice. Barplots showing average proportions of bacterial taxa present in (A) male mice and (B) female mice within a single cage. Bacterial sequence counts were rarefied to 36,481 sequences per sample and averaged across all of the mice of a given genotype within the same cage (two HET mice were housed with KO mice). The microbial community composition of each cage is represented by a single bar. The proportions of taxa are shown on the y-axis. The cage number and genotype of each community are shown on the x-axis separated by an underscore. Taxa present at less than 2% relative abundance were grouped into the “Other” category. The most specific taxonomic classification of the sequences is shown and the displayed taxon level is represented by a single letter code preceding the classification; o = order, f = family, g = genus.
(PDF)
