# Supplementary material for: The Znt7-null mutation has sex dependent effects on the gut microbiota and goblet cell population in the mouse colon
Source: PLoS One. 2020 Sep 29;15(9):e0239681. doi: 10.1371/journal.pone.0239681 (PMC7523961; doi:10.1371/journal.pone.0239681)
Supplement: S1 Fig — Actb was used as an internal reference for calculation of Muc2 fold difference using 2–ΔΔCt [23]. Values represent the average of technical triplicates for n = 4–5 mice/genotype/sex. Student’s t-test was used to compare values to the transcription level in male WT mice. No significant differences were found. (PDF) [file pone.0239681.s001.pdf]

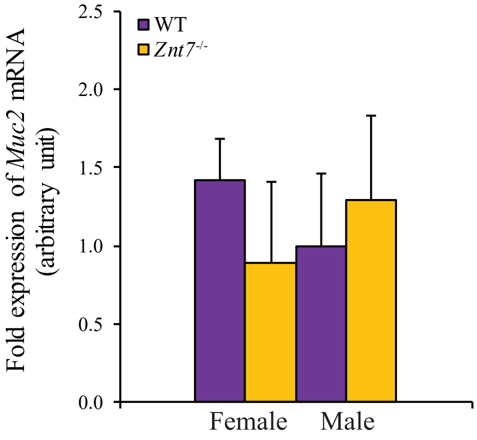

S1 Fig. mRNA expression of the *Muc2* gene in colonic tissue distal to the cecum. *Actb* was used as an internal reference for calculation of *Muc2* fold difference using $2^{-\Delta\Delta Ct}$ [23]. Values represent the average of technical triplicates for n=4-5 mice/genotype/sex. Student's *t*-test was used to compare values to the transcription level in male WT mice. No significant differences were found.
