# Supplementary material for: The Znt7-null mutation has sex dependent effects on the gut microbiota and goblet cell population in the mouse colon
Source: PLoS One. 2020 Sep 29;15(9):e0239681. doi: 10.1371/journal.pone.0239681 (PMC7523961; doi:10.1371/journal.pone.0239681)
Supplement: S2 Fig — Boxplots of the alpha diversity measured by (A) total observed sequence variants (SVs) or (B) Faith’s Phylogenetic Diversity are shown. There were no statistically significant differences between experimental groups for these measures. (PDF) [file pone.0239681.s002.pdf]

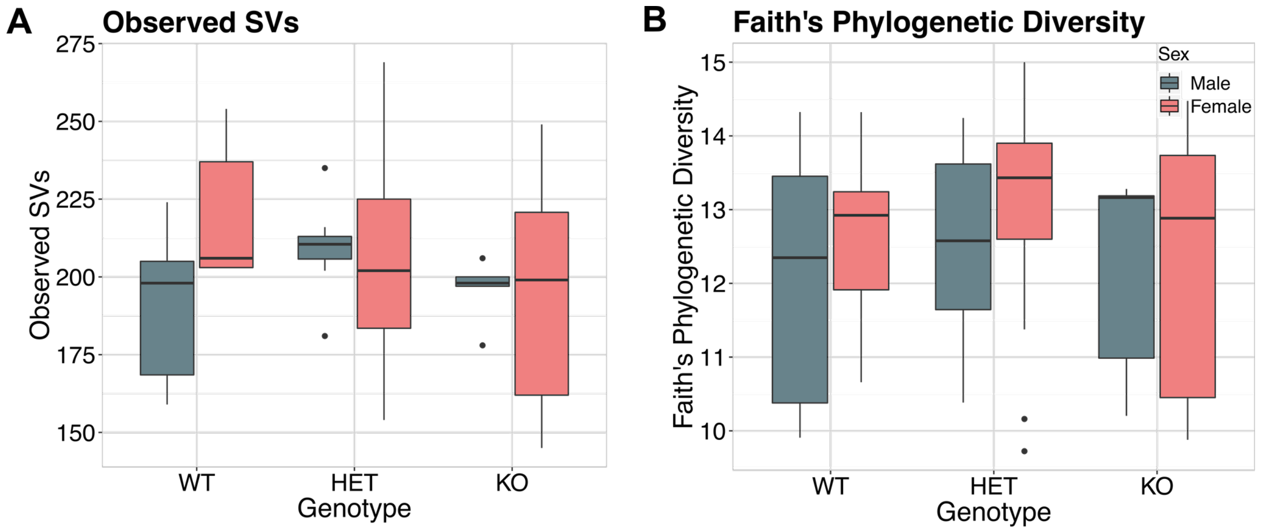

S2 Fig. Alpha diversity of mouse fecal microbial communities summarized by sex and genotype. Boxplots of the alpha diversity measured by (A) total observed sequence variants (SVs) or (B) Faith's Phylogenetic Diversity are shown. There were no statistically significant differences between experimental groups for these measures.
