# Supplementary material for: The Znt7-null mutation has sex dependent effects on the gut microbiota and goblet cell population in the mouse colon
Source: PLoS One. 2020 Sep 29;15(9):e0239681. doi: 10.1371/journal.pone.0239681 (PMC7523961; doi:10.1371/journal.pone.0239681)
Supplement: S3 Fig — The alpha diversity measurement of bacterial community evenness is shown (x-axes) relative to the proportions of (A) Allobaculum, rho = -0.8667347, p<2.2 x 10^−16^, (B) Bacteroidales, rho = 0.5289796, p<0.001, and (C) Clostridiales, rho = 0.5872449, p < 0.0001, (D) Lactobacillus, rho = -0.1709184, p = 0.2395 and (E) Helicobacter, rho = 0.1867347, p = 0.1983 are shown on the y-axis. A regression line showing the linear relationship between evenness and proportion of each bacterial taxa (Spearman) is shown on each graph. (PDF) [file pone.0239681.s003.pdf]

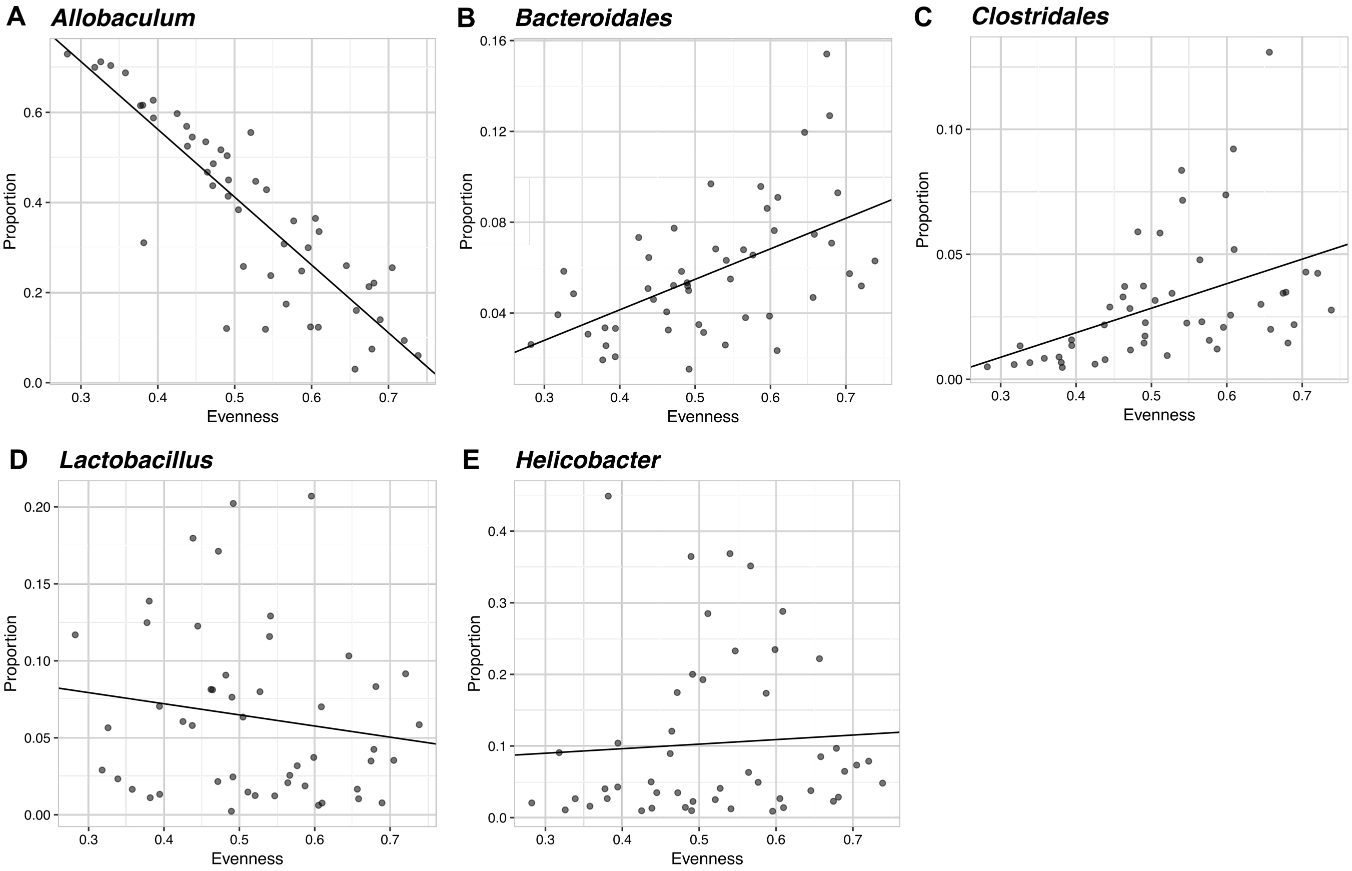

S3 Fig. Relationship between evenness and the relative proportion of abundant bacterial taxa within the mouse colon. The alpha diversity measurement of bacterial community evenness is shown (x-axes) relative to the proportions of (A) *Allobaculum*, rho = -0.8667347, $p<2.2 \times 10^{-16}$, (B) *Bacteroidales*, rho = 0.5289796, $p<0.001$, (C) *Clostridiales*, rho = 0.5872449, p < 0.0001, (D) *Lactobacillus*, rho = -0.1709184, p = 0.2395 and (E) *Helicobacter*, rho = 0.1867347, p = 0.1983 are shown on the y-axis. A regression line showing the linear relationship between evenness and proportion of each bacterial taxa (Spearman) is shown on each graph.
