# Supplementary material for: The Znt7-null mutation has sex dependent effects on the gut microbiota and goblet cell population in the mouse colon
Source: PLoS One. 2020 Sep 29;15(9):e0239681. doi: 10.1371/journal.pone.0239681 (PMC7523961; doi:10.1371/journal.pone.0239681)
Supplement: S4 Fig — Barplots showing average proportions of bacterial taxa present in (A) male mice and (B) female mice within a single cage. Bacterial sequence counts were rarefied to 36,481 sequences per sample and averaged across all of the mice of a given genotype within the same cage (two HET mice were housed with KO mice). The microbial community composition of each cage is represented by a single bar. The proportions of taxa are shown on the y-axis. The cage number and genotype of each community are shown on the x-axis separated by an underscore. Taxa present at less than 2% relative abundance were grouped into the “Other” category. The most specific taxonomic classification of the sequences is shown and the displayed taxon level is represented by a single letter code preceding the classification; o = order, f = family, g = genus. (PDF) [file pone.0239681.s004.pdf]

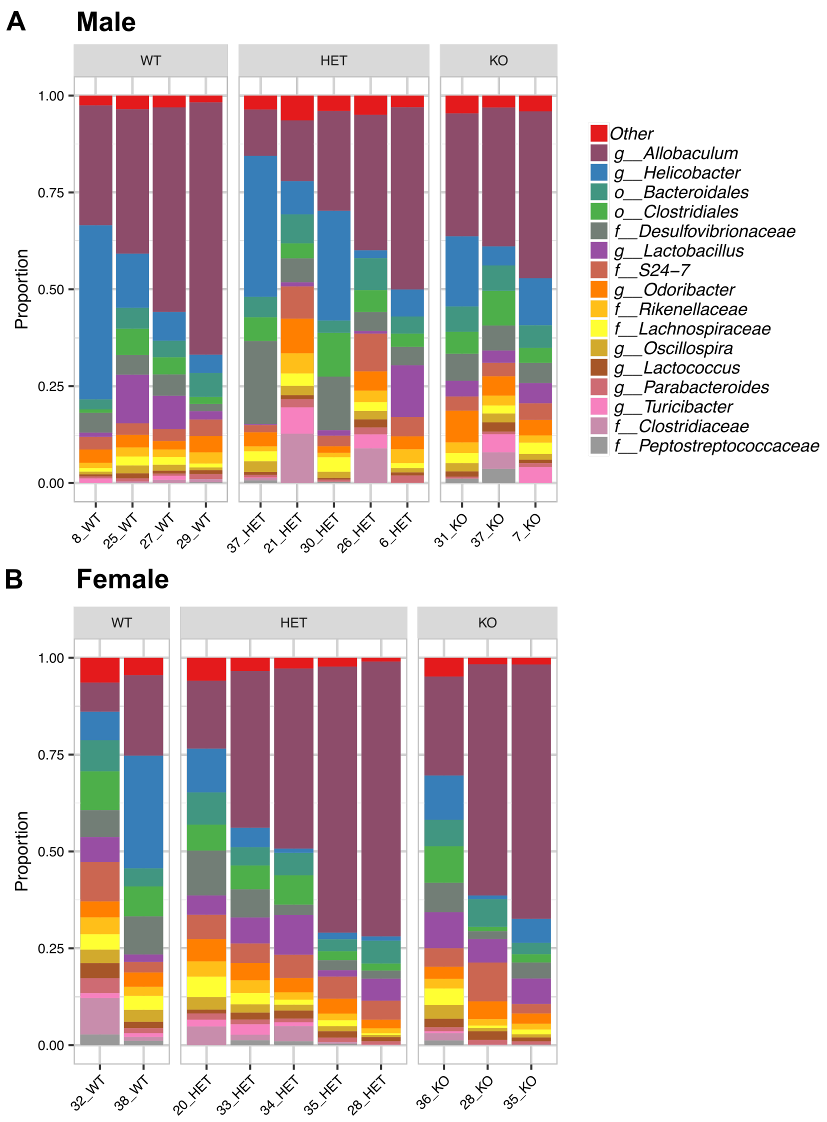

S4 Fig. Microbial communities averaged by cage show the same trends in *Allobaculum* abundance as individual mice. Barplots showing average proportions of bacterial taxa present in (A) male mice and (B) female mice within a single cage. Bacterial sequence counts were rarefied to 36,481 sequences per sample and averaged across all of the mice of a given genotype within the same cage (two HET mice were housed with KO mice). The microbial community composition of each cage is represented by a single bar. The proportions of taxa are shown on the y-axis. The cage number and genotype of each community are shown on the x-axis separated by an underscore. Taxa present at less than 2% relative abundance were grouped into the "Other" category. The most specific taxonomic classification of the sequences is shown and the displayed taxon level is represented by a single letter code preceding the classification; o = order, f = family, g = genus.
